# An Innovated Application of Reutilize Copper Smelter Slag for Cement-based Electromagnetic Interference Composites

**DOI:** 10.1038/s41598-018-34680-5

**Published:** 2018-11-01

**Authors:** Yong Fan, Bo Zhang, Jianxun Song, Vladimir Volski, Guy A. E. Vandenbosch, Muxing Guo

**Affiliations:** 1 Institut für Eisen- und Stahltechnologie, TU Bergakademie Freiberg, 09599 Freiberg, Sachsen Germany; 2 Department of Materials Engineering, KU Leuven, Kasteelpark Arenberg 44, B-3001 Heverlee, Leuven Belgium; 3 School of Metallurgy, Northeastern University, Shenyang, 110819 China; 4 Henan Province Industrial Technology Research Institute of Resources and Materials, Zhengzhou University, 450001 Zhengzhou, China; 5 Department of Electrical Engineering, KU Leuven, B-3001 Heverlee, Leuven Belgium

## Abstract

In the present study, it has been attempted an innovated application, *i*.*e*., electromagnetic interference shielding material, to reutilize copper smelter slag, aiming at an alternative high value added product. Notably, a proof-of-concept experiment with an addition of a 45 wt.% of copper slag alone to the cement matrix boost the shielding effectiveness (SE) to approximately 7–8 dB in the 500 MHz–1.5 GHz frequency range, highlights the incident electromagnetic wave has been weakened by approximately 60 pct. This phenomenon is attributed to the iron silicate, fayalite, and magnetite embedded in the sample mixture serve as magnetic and dielectric loss absorbent, deriving from the copper slag. Copper slag with low value application, shows its competitive economic and social advantages as candidate infill for electromagnetic interference shielding materials.

## Introduction

Smelting is the pyrometallurgical process used to produce copper metal with the use of mine concentrates or copper scrap as the primary source of feed. In recent years, world copper production has reached nearly 19 million tonnes copper. Flash-continuous technology is used in more than half of all refineries (about 70%), which is expected to remain this level until 2019^1^. In the flash smelting process, a “matte” is formed containing 50–70 mass% copper. It is developed in a converter to produce a “blister copper” with 98.5–99.5 mass% copper. Then, the blister copper is refined, and followed by casting into anodes for electro-refining^2^. In general, during the above smelting process, two kinds of slags are generated. One is smelter slag, and the other is converter slag. In practice, copper converter slag is recharged back to flash smelter as copper concentrate. During the whole smelting process, copper smelter slag, nearly 30 million tons produced every year in the world, is outflowed for recycling, such as metal recovery, production of the high-value added products and for disposal in slag dumps or stockpiles^3^ One the other hand, the recent overcapacity of the cement industry (dominating consumer of copper slag) in the developed markets has been causing increasing concern, thereby, urgently necessitating the other utilization of slag^4^. There are some applications using copper slags such as concrete aggregates^5^ abrasives^6^ ceramic tiles^7^ and land reclamation^8^ *etc*. In the present work, it has been attempted to seek an innovated application, *i*.*e*., electromagnetic interference shielding material, aiming at high valued added product.

Electromagnetic interference (EMI) shielding presents enormous interest due to the increasing abundance, density and sensitivity of electronics^9^. It is also required for securit concerns on wireless communications. Furthermore, there are worries in the biological threats that might (not explicit) arise due to exposure to EM fields, especially for children^10^. Cement based composites are the most common structural materials used in constructions. Accordingly, many researchers^11^ have investigated the EMI shielding properties of cement through introducing “infills,” for instance, carbon^12^ metal^13^ and ferrite^14^. In general, materials with high conductivity such as carbon or metal are applicable to be as infills^15^ due to their free electrons, which could act as mobile charge carriers interacting with the EM field, causing the reflection of the radiation^16^. Moreover, high magnetic permeability materials such as hematite, magnetite or other ferrites^17^ could provide magnetic dipoles, which interact with the EM fields, and resulting in the absorption of the radiation. Cao and Chung^18^ have observed that the fly ash applied into cement composites can strengthen the absorption of the radiation. Fan. etc^19^ have also reported that the metal droplets and ferrite contained in the steel furnace dust, which mixed in the cement matrix, raises its EMI shielding effectiveness of the composite. Although the shielding effectiveness of these recent attempts is low compared to that of commonly used carbon-based infills^20^ industrial waste is much lower in cost as their advantages, together with socially positive effectiveness.

During the copper smelting process, copper-rich matte (sulphides) and copper slag (oxides) are formed as two separate liquid phases due to the addition of silica, forming strongly bonded silicate anions by combining with the oxides. The molten slag is discharged from the furnace at 1273–1573 K. In most cases, a quick solidification by pouring molten slag into water gives a granulated amorphous slag^21^. This slag contains iron silicate, fayalite, and magnetite that could be a possible infill applied to EM shielding, which provides a practical and value-added way for the slag reutilization and recycling.

## Experimental Method

Table 1 shows the composition of the granulated amorphous copper slag, which was supplied by a copper smelter. The chemical compositions were analyzed by inductively coupled plasma optical emission spectroscopy (ICP-OES, Varian 720 ES, USA). The mineral phases and morphology were analyzed by X-ray diffraction (XRD, 40 kV, 30 mA, Cu-Kα, SEIFERT 3003 T/T, Germany), scanning electron microscope and energy dispersive spectrometer (SEM-EDS, XL30FEG, Philips, USA), and electron probe microanalysis (EPMA, JXA-8530F, Jeol, Japan).

**Table 1:** The chemical composition of the copper smelter slag (wt.%).

TFe	SiO_2_	CaO	Al2O3	MgO	Cr	Zn	Cu
41.5	32.1	0.7	4.5	0.5	0.3	6.8	0.2

The copper slags with different addition levels of 15, 30 and 45 wt.% were mixed into a typical Portland cement. Water was added into the mixture of cement and copper slag to achieve the moisture content of approximately 30 wt.%. Then the mixtures (200 g) were shaped within a Teflon mold with dimension of 140 mm inside length and dried for 48 hours in the fuming cupboard. Finally, the specimens with 5 ± 0.3 mm thickness were prepared after demolding and curing at room temperature and humidity. To compare the shielding effectiveness, magnetite powders (Fe_3_O_4_, >99.5 pct, Sigma-Aldrich, USA), hematite powders (Fe_2_O_3_, >99 pct, Sigma-Aldrich, USA), and metallic iron powders (Fe, >99.5 pct, Emsure, Germany) were respectively used as filler with the addition level of 15 wt.%.

**Figure 1:**
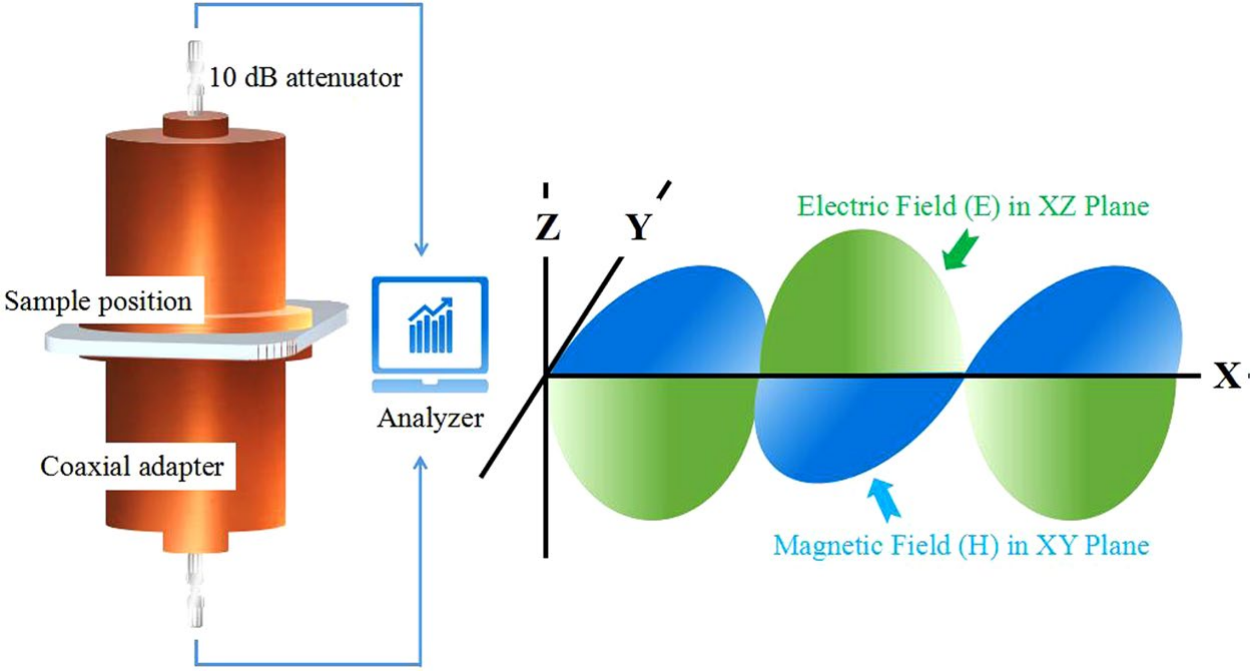
The SE test fixture and its electric and magnetic field distribution.

Figure 1 shows the shielding effectiveness test fixture EM-2017A, which is composed by two coaxial adapters, two attenuators (10 dB, 50 Ω, fixed on each end of the adapters), and a spectrum analyzer (Keysight N9344C) with a built-in tracking generator. By analyzing the consecutive transmission measurements between the top and bottom adapters with and without the specimen present, the shielding effectiveness of the specimens was evaluated^22^.

## Results and Discussion

### Characterization of the copper slag

The compositional analysis (Table 1) confirms the presence of iron, silicon, zinc, and aluminum as the significant element in the copper slag, and some other elements in lower level of concentrations, such as calcium, magnesium, chromium and copper. This waste is regarded as hazardous materials in many countries due to the presence of heavy metals. The environmental evaluation are beyond the scope of the current study, however, more attention would have to be paid to that.

**Figure 2:**
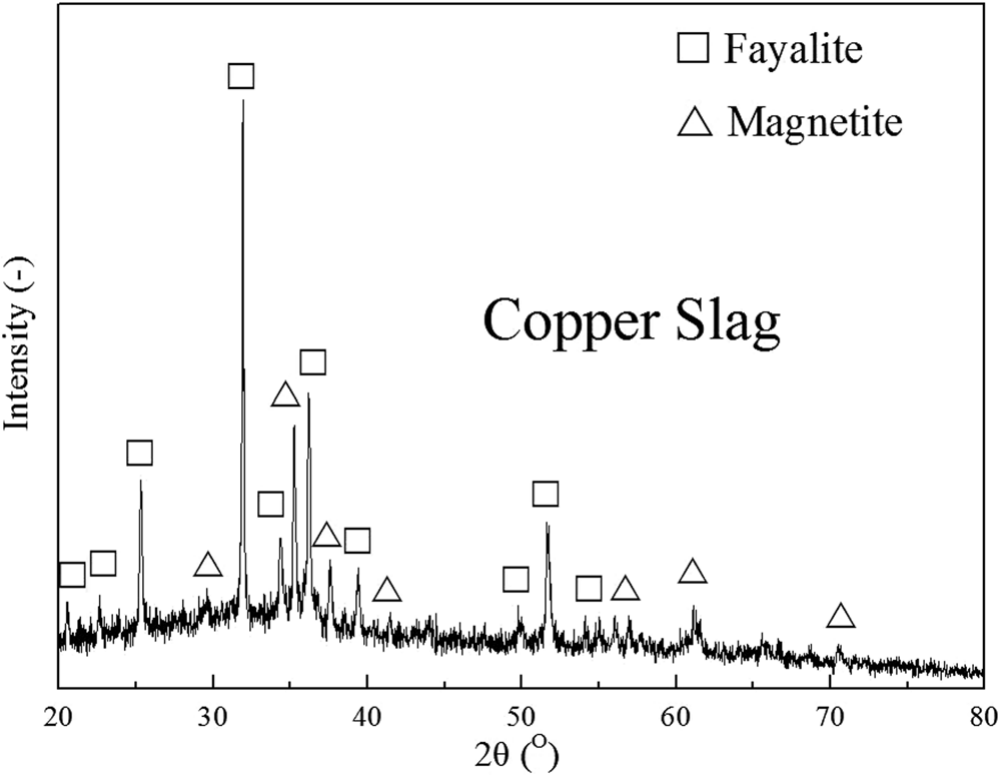
X-ray diffraction (XRD) patterns of the copper slag.

**Figure 3:**
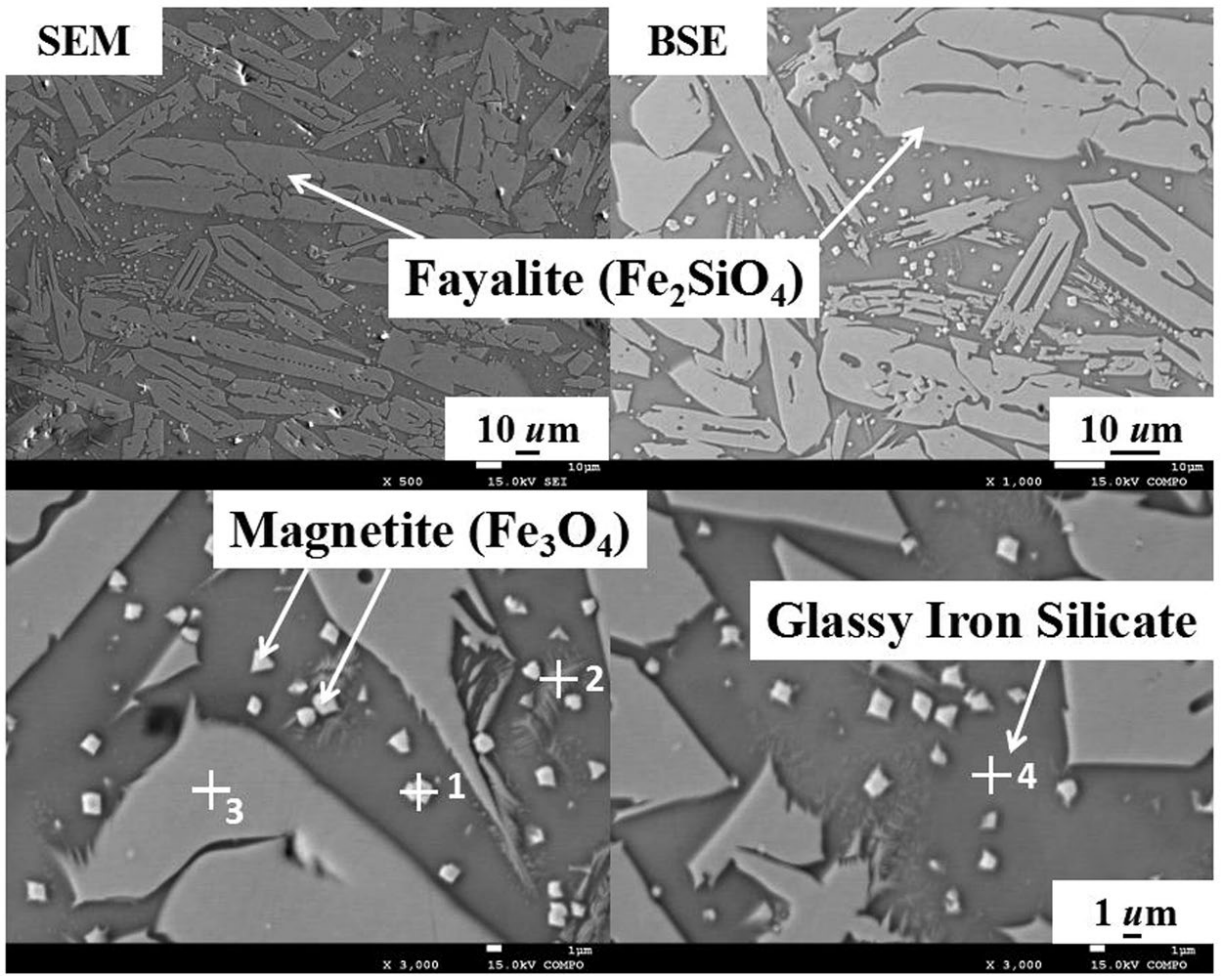
Scanning Electron Microscopy(SEM) image and Back Scattered Electron (BSE) images of copper slag particulates.

As seen in XRD patterns (Fig. 2), SEM and BSE images (Fig. 3) and point analysis (Table 2), iron silicate was the main component of the water-granulated copper slag, which was the base of slag matrix. Iron is mostly crystallized as fayalite (Fe_2_SiO_4_) and magnetite (Fe_3_O_4_) during the matte smelting process or water cooling. There are some characteristic morphologies of crystal phases could be observed. The magnetite shows a cubic shape crystal with approximately 1 um diameter (Fig. 3, phase 1), while the fayalite (with a high content of aluminum and zinc) presents as a spindle like crystal, which has thin and long branches (Fig. 3, phase 2 & 3), distributed in the glassy matrix (Fig. 3, phase 4). Other trace elements or phases are presented which we will not go further in details in the current paper.

**Table 2:** Point analysis of typical phases of copper slag particulates in Fig. 3 (wt.%).

	Fe	O	Al	Si	Ca	Zn	Cr
1	52.1	27.3	5.1	5.0	—	6.9	1.5
2	33.4	33.3	4.0	18.8	1.1	7.2	—
3	50.5	28.5	—	13.4	—	5.8	—
4	40.3	32.8	2.4	14.9	0.6	6.6	0.3

### Microstructure of the Cement-based composites

**Figure 4:**
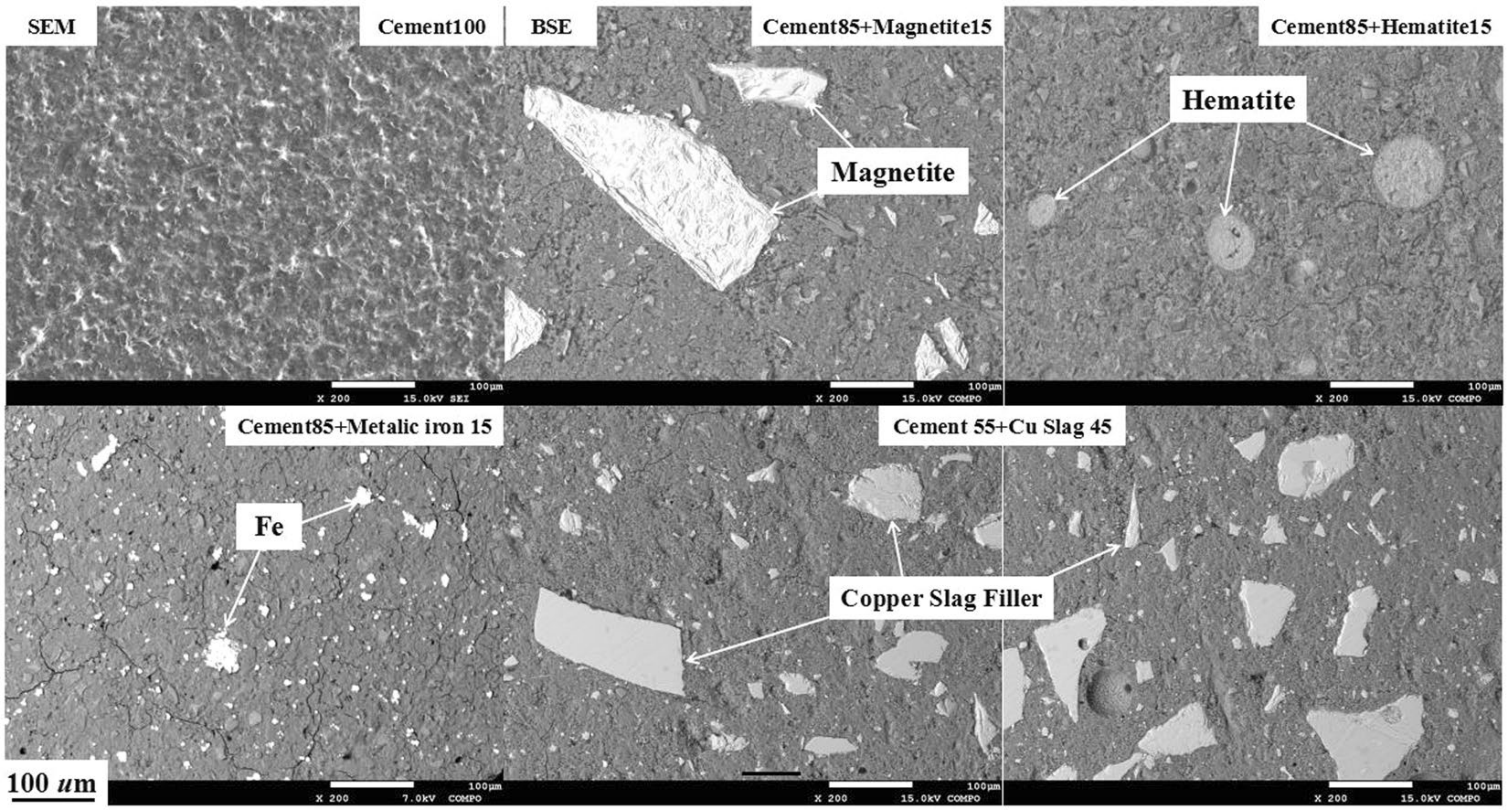
SEM and BSE images of references and the composite with 45 wt.% of copper slag.

The microstructure of reference samples shown in Fig. 4 are pure cement (Cement100), 15 wt.% magnetite (Cement85+ Magnetite15) and hematite (Cement85+ Hematite15) infilled cement-based composites, and the composite with 45 wt.% of copper slag synthesized with cement (Cement55+ Cu Slag45), respectively. Characteristically, references and copper slag could be found evenly dispersed in these samples, which were then measured the EMI shielding effectiveness in a series for comparison.

### EMI shielding effectiveness and mechanism

The EMI shielding effectiveness (SE) of a shielding material defined as a ratio of the transmitted to the incident energy. The shielding of an EM wave happens with three mechanisms which are reflection loss (at the surface), absorption loss (through the shield), and multiple internal reflections (interior of the shield). Total SE is a summation of shielding by these three mechanisms at the surface and interior of the shield. It is performed in the frequency range of 500 MHz–1.5 GHz for the coaxial transmission line measurement. It is considered that these frequency ranges are very critical to commercial applications, such as television, wireless network, mobile phone, *etc*.

**Figure 5:**
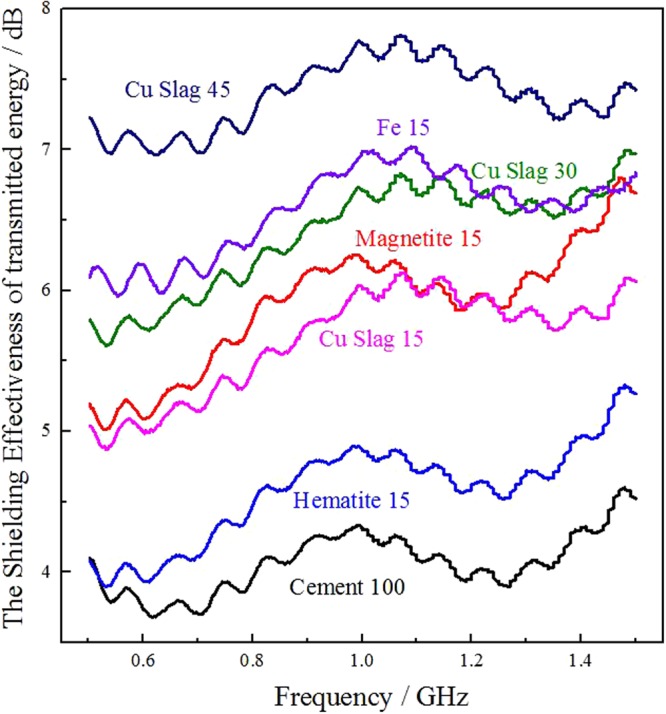
The shielding effectiveness of references and copper slag filled samples of 5 mm thickness.

The measured shielding effectiveness of the references and copper slag filled samples with 5 mm thickness was shown in Fig. 5. Generally, the shielding effectiveness of material is related to the electric conductivity and the EM parameters of its composite. The cement matrix is inert in conductivity, showing a relatively low shielding effectiveness (Fig. 5, Cement100). Typical magnetic loss absorbent such as magnetite and hematite would absorb EM wave by polarization mechanisms, which shows a robust in SE of the composite (Fig. 5, Magnetite15 & Hematite15). Metal is an impressive dielectric loss absorbent, which would constrict EM energy by electronic and ionic polarization. Metallic iron filled sample illustrated a higher EM shielding effectiveness (Fig. 5, Fe 15) than that of cement 100, Magnetite15 and Hematite15. The sample with copper slag as an infill, namely 15 wt.% (Fig. 5, Cu Slag15), 30 wt.% (Fig. 5, Cu Slag30), and 45 wt.% (Fig. 5, Cu Slag45) indicated an intensification of the shielding effectiveness. Notably, the addition of a 45 wt.% of copper slag alone to the cement matrix boost the shielding to approximately 7–8 dB in 500 MHz–1.5 GHz, highlights the incident EM wave has been weakened by approximately 60 pct. This phenomenon is attributed to the fayalite and magnetite embedded in the sample mixture serve as magnetic and dielectric loss absorbent, which derives from the copper slag.

Ferrites are the prevalent infills used in cement based composites. Literally, reflection cannot lessen or depress radiation, and the reflected wave may interact with the incident wave, which might matter other units or devices. With the service of EM absorbing materials transferring the energy to other forms, the EMI radiation could be vitiated to the furthest. Cao and Chung^18^ have prepared the fly ash filled cement composite with EMI SE of approximately 4 dB (4.3 mm thick, 1.0–1.5 GHz). This suggests that the existence of hematite in the fly ash can enhance shielding through absorption. The ferrite contained in the copper slag are exceptional choices of wave absorbing component for cement based EMI shielding materials.

**Figure 6:**
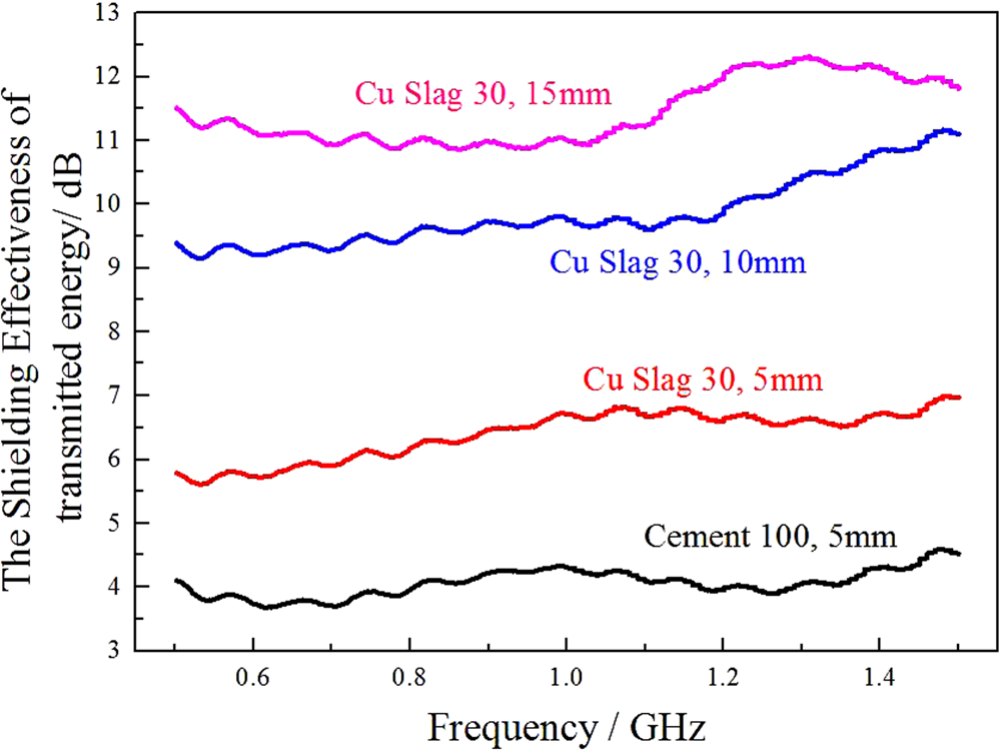
The shielding effectiveness of copper slag filled sample in different thickness.

Figure 6 shows the influence of sample thickness on EM shielding of the copper slag infilled materials. It is clear that the SE level of the samples is reinforced with the increase of thickness. In reality, the thickness of the shield is a crucial economic factor in the future application. Copper slag with low value application, shows its competing preference in some application, for example, massive underground or remote information base construction. Moreover, the reutilization of copper slag has its social significance and benefit.

Copper slag is usually valorized by extracting metals or used to produce some construction materials (low valuable added). The present study has attempted to apply it to the cement matrix for obtaining EMI shielding function, which provides practical and high added-value reutilization. Furthermore, not only by adding the slag up directly to cement, but also through metallurgical modification, such as carbothermal reduction, magnetization roast, or controlled molten oxidation^23^ the EMI of the product prepared with that modified copper slag may be intensely improved. A new direction for the multidisciplinary cooperation of metallurgical, recycling, and electromagnetic field is provided in these emerging researches.

## Conclusions

The present study attempts to apply copper slag to the cement matrix for obtaining EMI shielding function, which provides a practical and high added value approach for its reutilization. Notably, the addition of a 45 wt.% of copper slag to the cement matrix boost the shielding value to approximately 7–8 dB in 500 MHz–1.5 GHz, highlights the incident EM energy has been weakened by approximately 60 pct. This phenomenon is attributed to the fayalite and magnetite deriving from copper slag embedded in the sample mixture, which serves as magnetic and dielectric loss absorbent. Moreover, with the increase of thickness, the SE level of sample is reinforced. Copper slag with low value application, shows its competing preference in some application, for example, massive underground or remote information base construction. Moreover, the reutilization of copper slag has its social significance and benefit.
